# False memories from nowhere: Humans falsely recognize words that are not attested in their vocabulary

**DOI:** 10.3758/s13423-025-02677-7

**Published:** 2025-03-14

**Authors:** Daniele Gatti, Marco Petilli, Michela Marchetti, Tomaso Vecchi, Giuliana Mazzoni, Luca Rinaldi, Marco Marelli

**Affiliations:** 1 Department of Brain and Behavioural Science, University of Pavia, Piazza Botta 6, 27100 Pavia, Italy; 2 Department of Psychology, University of Milano-Bicocca, Milan, Italy; 3 Department of Health, Dynamic and Clinical Psychology, University of Sapienza, Rome, Italy; 4 Cognitive Psychology Unit, IRCCS Mondino Foundation, Pavia, Italy; 5 Department of Psychology, University of Hull, Hull, UK; 6 Neuromi, Milan Center for Neuroscience, Milan, Italy

**Keywords:** DRM, False memory, Pseudowords, Distributional semantics, Semantic memory

## Abstract

Semantic knowledge plays an active role in many well-known false memory phenomena, including those emerging from the Deese–Roediger–McDermott (DRM) task. Indeed, in this experimental paradigm, humans tend to falsely recognize newly presented words via activation of other previously shown stimuli. In the present study we aimed to test what happens in cases in which no apparent prior semantic knowledge is available, like in the case of entirely novel lexical stimuli. To do so, we evaluated semantic similarity effects in a DRM task with lists entirely composed by pseudowords (or “novel words,” i.e., letter strings resembling real words but lacking assigned meanings). Semantic similarity between pseudowords were established through a distributional semantic model able to represent in a vector space, not only attested words but also unmapped strings as bags of character n-grams. Participants were instructed to memorize those lists and then to perform a recognition task. Results showed that participants false and veridical recognition increased with increasing semantic similarity between each stimulus and the stimuli comprising its list, as estimated by the distributional model. These findings extend previous evidence indicating that humans are sensitive to the semantic (distributional) patterns elicited by novel words by showing that this sensitivity can even induce humans to falsely recognize stimuli that they have never encountered in their entire lives.

## Introduction

Semantic memory is an active, generative system (e.g., Jones et al., 2015; Kumar, 2021) enabling humans to assign labels to novel entities, either by combining two existing words (e.g., “smartphone” as smart + phone), manipulating existing ones (e.g., “pessimize”), borrowing terms from other languages (e.g., “agelast” from the Greek “agélastos”: not laughing) or even creating entirely novel strings of letters (e.g., “hobbit” from J.R.R. Tolkien’s books).

At a more implicit level, these generative abilities also characterize well-known memory phenomena, like false memory for items semantically related to the ones previously encoded. That is, starting with Bartlett’s (1932) and Roediger and McDermott’s (1995; but see also: Brainerd et al., 2008) pioneering studies, it has been shown that a moderate number of memory distortions can be explained in terms of reliance on prior (semantic) knowledge. These distortions are related to humans’ tendency to prioritize the extraction of the gist of the encoded information (i.e., the meaning) over its precise features (e.g., Brainerd et al., 2020; Sulin & Dooling, 1974).

Here, we aimed to probe the limits of semantic-memory generative properties by exploring cases in which no apparent semantic knowledge is available, like in the case of entirely novel stimuli. Examples for these never attested stimuli are strings of letters (often defined as “novel words” or “pseudowords”) that are consistent with the orthotactical rules of a given language, but do not appear in the written or oral language (e.g., “boppies” or “quocky”), and thus are not conventionally associated with a given meaning. When asked to encode and recognize, indeed, one could assume that humans would rely on purely episodic processes.

Nevertheless, the idea that, when dealing with pseudowords, our memory system would act as a *tabula rasa* has been challenged by several studies. That is, even though these stimuli have no place in a given vocabulary, they can predictably activate semantic memory (e.g., Cassani et al., 2020; Chuang et al., 2021; Gatti et al., 2023a, 2024a, 2024b; Hendrix & Sun, 2021; Josse et al., 2024). It is indeed possible to quantify the semantic information triggered by these strings of letters through distributional semantic models (DSMs). Briefly, DSMs represent word meanings as high-dimensional numerical vectors induced from large corpora of natural language under the assumption that the contexts in which words occur can approximate their meanings (Harris, 1954; Wittgenstein, 1953). Thus, words with similar meanings will be mapped to nearby points in a semantic space (Günther et al., 2019; Mandera et al., 2017). Expanding on this, DSMs can be used to retrieve a representation for stimuli not attested in the training corpus by modelling them on the basis of the sequences of *n* contiguous letters (labeled as n-grams) composing it, that is by quantifying the distributional patterns of their sublexical elements (Bojanowski et al., 2017). This approach has been used to approximate the “meaning” of pseudowords, that is, the semantic patterns that an unfamiliar letter string can elicit.

However, existing studies have limited their investigation to the contingent relationship between the task that participants perform and the (semantic) information that pseudowords elicit. For example, previous studies showed that humans are sensitive to the semantic patterns evoked by pseudowords in tasks in which the response to a given stimulus is collected while that same stimulus is being processed, like in the case of lexical decision (e.g., Bonandrini et al., 2023; Hendrix & Sun, 2021). Conversely, no evidence is available regarding humans’ behavior in tasks in which (i) encoding and long-term storage of subword semantic information is involved and (ii) previous encoding of subword semantic information exerts influence on subsequent recognition memory. Thus, in the present study, to further investigate how humans encode, store and use prior distributional information when dealing with novel information, here we took advantage of DSMs paired with the most widely used tool to investigate recognition memory and distortions, that is the Deese–Roediger–McDermott task (DRM; Deese, 1959; Roediger & McDermott, 1995). Briefly, in the DRM task, participants are asked to encode lists of stimuli and then, after a distracting task, they are asked to perform a recognition task. The items composing each list are (semantically) related (but see Coane et al., 2021, for a comprehensive review of the various similarities influencing human memory in the DRM task) to a non-shown target item, named critical lure; e.g., *bed*, *rest*, *awake*, *tired*, *dream*, etc. – critical lure: *sleep*. During the recognition phase, participants falsely recognize critical lures as if they were part of the memorized lists, although these items were never presented during the encoding phase.

To test whether the semantic information carried by pseudowords can trigger the DRM effect, and thus if humans falsely recognize completely unfamiliar stimuli (i.e. unattested not only in the encoding phase of the task, but in the entirety of their past experiences), in the present study we generated DRM lists composed by pseudowords and then asked participants to perform a recognition task. In this latter phase, participants were shown studied items as well as pseudowords that were semantically related to the studied ones, according to our computational model. Mimicking what happens for words (Gatti et al., 2022), we expected participants’ proportion of (veridical and false) recognition for each pseudoword to be predicted by the semantic similarity between the stimulus and the set of studied ones.

## Methods

### Participants

Sixty-three students participated in the study (34 females, *M* age = 26.25 years, *SD* = 3.36, age range = 21–35 years). All participants were native Italian speakers, had normal or corrected-to-normal vision, and were naïve to the purpose of the study. Informed consent was obtained from all participants before the experiment. The protocol was approved by the psychology ethics committee of the University of Pavia (Italy) and participants were treated in accordance with the Declaration of Helsinki.

Sample size was determined a priori by means of a data simulation procedure. We chose to include as estimate for the effect size a value that was around one-third of the one observed in a recent study using DRM on words (Gatti et al., 2023b), in which it was observed as a standardized effect size in the multilevel logistic regression a *β* = 0.94. Here, we used *β* = 0.30. The choice to use this value was driven by the fact that we expected the semantic effect elicited by pseudowords to be smaller as compared to words (see Gatti et al., 2023a). The simulation showed that the analysis employed here reached a power of 80% when including at least 58 participants.

### Distributional semantic model

The DSM used here was *fastText* (Joulin , 2016). *FastText* is based on the idea of taking into account subword information by inducing semantic representations as the average vector of the letter n-grams associated with each word (Bojanowski et al., 2017; Schütze, 1992). This solution solves not only issues related to the often low-quality representation of infrequent words but also allows to retrieve semantic vectors for strings of letters not attested in the training corpus. That is, while classical DSMs were bounded to the information learnt in the training phase, *fastText* models overcome their limitations and, ultimately, allow for the representation of pseudowords by relying on the distribution of their n-grams.

**Fig. 1:**
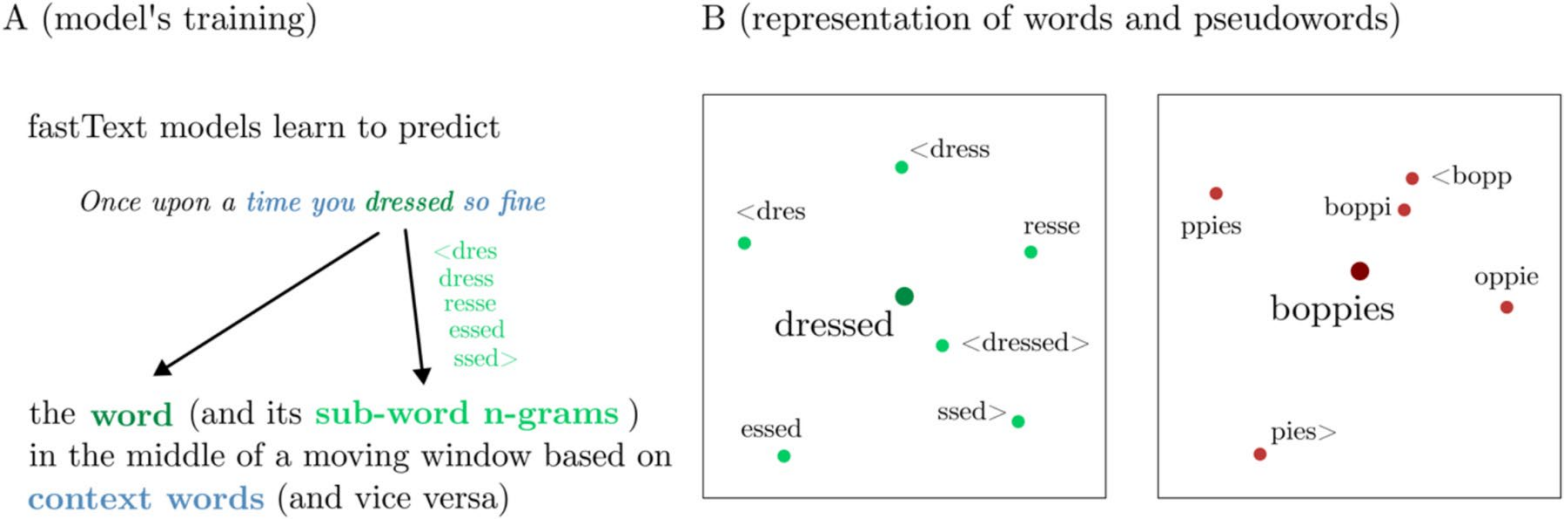
Graphical representation of how *fastText* computes word and sub-word vectors (**A**) and practical examples of how the vectors of the example word “dressed” (bigger green dot) and pseudoword “boppies” (bigger red dot) are computed as averaged vector (i.e. the centroid) of its embedded n-grams (light green or light red dots, respectively) (**B**). Note that < and > respectively represent the starting and ending strings

The model used was trained on Common Crawl (around 630 billion words) and Wikipedia (around 9 billion words) using the continuous bag of words method (Mikolov et al., 2013), with 300 dimensions, a co-occurrence window of five words and n-grams of length 5 (Grave et al., 2018). As an example, consider the word “dressed”, composed by several 5-g. The *fastText*-induced representation will be the sum of the vector for the word < dressed > along with the vectors of the (closed) ngrams < dres, dress, resse, essed, and ssed > , which is then divided by the number of vectors included in the sum (7, in our example) (Fig. 1B, left part). Note that starting and ending strings (i.e., < and >) are encoded as independent characters and thus < dres and ssed > count as 5-g.

A similar approach can be applied to unattested strings in order to capture the semantic information associated with pseudowords.^1^ Let’s take as an example the (pseudo-English) string *boppies* (see Fig. 1B, right part). Of course, in this latter case the induced representation will not consider the < boppies > vector (since it does not exist by itself), but only its embedded n-grams, like for example: < bopp, boppi, oppie, ppies, and pies > . That is, even though the model did not encounter the string < boppies > , it can extract information from the distribution of its embedded subwords in natural language.

Using *fastText*, we therefore obtained semantic representations for the pseudowords included in this study.^2^ For each pair, we computed a semantic-relatedness index based on the cosine of the angle formed by vectors representing the meanings of the corresponding strings. The higher the semantic-relatedness value, the more semantically related the letter strings are expected to be, as estimated by the model.

### Stimuli

The DRM lists used were automatically constructed using the False Memory Generator (FMG) tool (Petilli et al., 2024). FMG is an automated and data-driven tool for generating DRM lists by exploiting similarity relationships among items populating a given vector space.

Specifically, starting from the Italian words included in the ANEW (Montefinese et al., 2014), using Wuggy (Keuleers & Brysbaert, 2010) we first obtained a large pool of pseudowords (> 100,000). Wuggy is a pseudowords generator that creates orthographic strings that respect the orthotactic rules of a given language. After removing duplicates, pseudowords not readable in Italian and pseudowords ending with recognizable Italian suffixes, we obtained 65,344 unique pseudowords. Then, using *fastText* we retrieved the vector representations for all the pseudowords. Using FMG on this vector space we then constructed 15 lists of 12 pseudowords each (180 studied pseudowords in total). Briefly, FMG establishes relationships between items by leveraging the similarity between their representations in a vector space, thus enabling the creation of multiple DRM lists. Notably, this removes the risk of some stimuli being associated with multiple lists. Firstly, FMG partitions the space in *k* clusters, with *k* = number of lists needed. Then, within each cluster, FMG further divides the space into a close and a far section, with respect to the centroid of the cluster, and selects “studied” (from the close section) and “new” (from both sections) items based on the user’s input details (i.e., type of distribution for new items, number of studied and new items). The convenience of the division into close and far space is threefold: it allows us (i) to have lists composed by studied items sampled from comparable sections of the space, (ii) to manipulate how many new items to sample from a section of the space overlapping with the one from which the studied ones are sampled, and more importantly, (iii) to avoid the overlapping between items composing different lists (i.e., when selecting items for a given list, the sampling cannot involve the close spaces of the other lists).

**Fig. 2:**
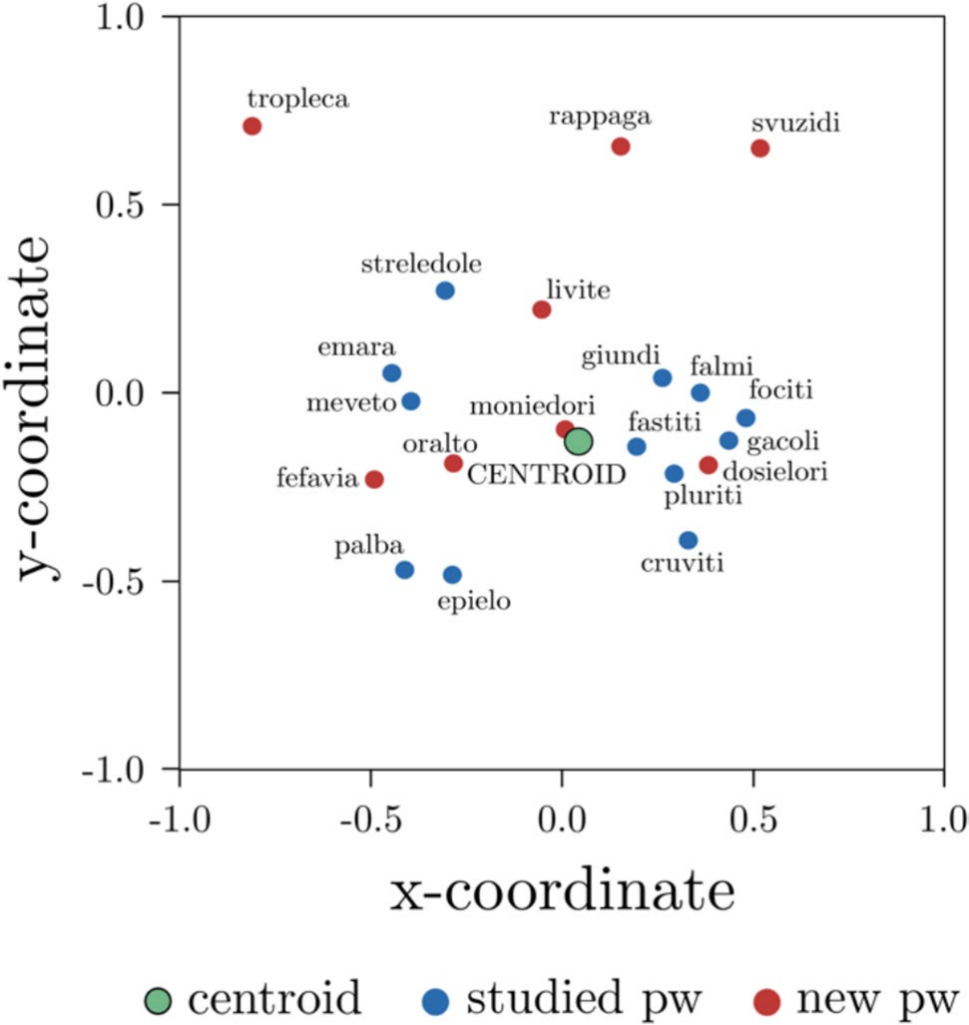
Scatterplot representing a sample list as resulting from an isoMDS procedure (i.e., a procedure that, given a matrix of distances among items, provides their two-dimensional coordinates; Venables & Ripley, 2002); studied pseudowords are represented in blue, new pseudowords in red and the centroid of the studied pseudowords in green

In the stimuli selection that we used, the distribution of the semantic similarity between each pseudoword and the centroid of the list (i.e., the mean vector of the studied items) had a continuous (i.e., Distributed Method in FMG) distribution (see Fig. 2 for a graphical representation). Thus, with such a distribution setting, for each list, we obtained eight pseudowords to be shown in the recognition phase along with four studied pseudowords. Additionally, all the pseudowords included in each list had an orthographic distance (Levenshtein distance, which quantifies the minimum number of single-character edits required to change one element into the other) larger than 2 with each other.

The recognition phase of each list was then composed of 12 pseudowords, four of which had been presented in the previous phase (i.e., studied pseudowords) and eight of which had not been previously presented (i.e., new pseudowords). Notably, differing from classical DRM tasks in which only one item is included as critical lure, here we adopted a broader perspective, manipulating in a continuous fashion the similarity between the new items and the studied items (see Petilli et al., 2024 for a discussion and the left panel of Fig. 4 for a graphical representation). The four studied pseudowords presented in this experimental phase were randomly selected from the studied lists.

### Procedure

Participants were tested using Psychopy (Pierce et al., 2019) through the online platform Pavlovia (https://pavlovia.org/). The task was divided into 15 blocks (one for each list, presented in random order). For each block, the participants had to memorize 12 pseudowords shown in descending semantic similarity with respect to the centroid of the list. Each trial started with a central fixation cross (presented for 500 ms) followed by a pseudoword (presented for 1,500 ms) and a blank screen (presented for 300 ms), then the script moved automatically to the next fixation cross. At the end of the encoding phase, participants were required to perform an attentional task (i.e., a modified version of the go-no-go) as a distracting task for 2 min. Then participants were asked to perform the recognition phase. In the recognition phase, participants were shown one pseudoword at a time and were instructed to respond if the pseudoword showed was old or new with respect to the 12 pseudowords belonging to the list studied in that block. Participants were asked to respond as fast and as accurately as possible by pressing two buttons of a standard keyboard (i.e., A and L) using their left and right hand; the response keys assignment was counterbalanced among participants. Each trial started with a central fixation cross (presented for 500 ms) followed by a pseudoword (presented until response); after participant’s response, a blank screen (lasting 1,000 ms) was presented and then the next trial began. This procedure (encoding task, distractor task, recognition task) was repeated for each of the 15 lists.

## Data analysis and results

**Fig. 3:**
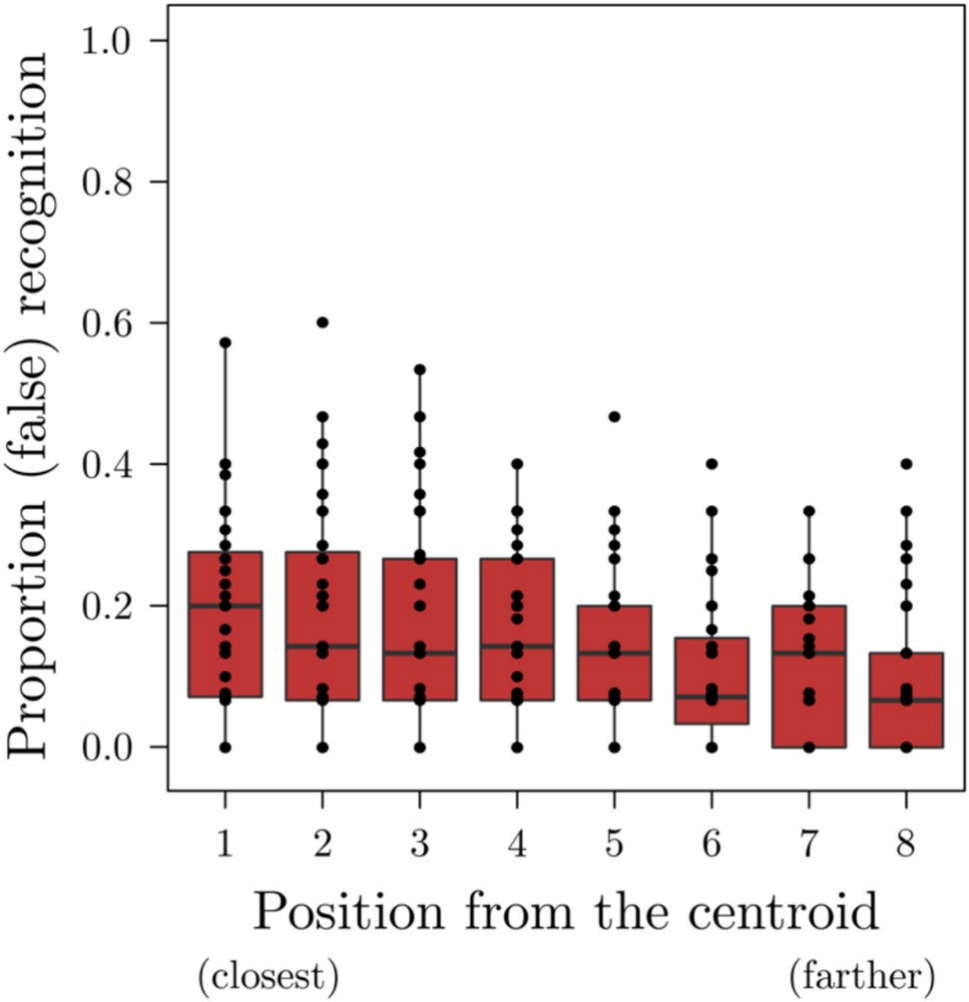
Boxplot illustrating participants’ proportion of old responses over the various new pseudowords from the closest (on the left) to the farther (on the right) from the centroid

Participants accuracy proportion was overall high, 0.80 (*SD* = 0.11) for studied pseudowords and 0.85 (*SD* = 0.8) for new ones. Regarding this latter category, given the structure of the stimuli (i.e., new items are distributed over a continuous range of similarity), in Table 1 we included mean proportion of old responses, standard deviations and ranges over the various items from the closest to the centroid to the farther aggregated by participant (see also Fig. 3 for a graphical representation).

**Table 1:** Descriptive statistics of the proportion of false alarm for each new item divided based on their position from the centroid aggregated by participants

Position from the centroid (new pseudowords)	Mean	SD	Range
1 (closest)	0.20	0.12	0–0.57
2	0.18	0.14	0–0.60
3	0.17	0.14	0–0.53
4	0.15	0.11	0–0.40
5	0.13	0.10	0–0.46
6	0.10	0.09	0–0.40
7	0.11	0.09	0–0.33
8 (farther)	0.09	0.09	0–0.40

All the analyses were performed using *R*-Studio (RStudio Team, 2015). Data were analyzed through a mixed-effects approach, which incorporates both fixed-effects and random-effects (associated to participants and task stimuli) and allows for the specification of predictors at both participants and/or item level. Multilevel logistic models were run using the *lme4 R* package (Bates, et al., 2015). Trials in which overall reaction times were faster than 300 ms or slower than 5,000 ms were excluded from the analysis (3% of the trials excluded). All models included participants and items as random intercepts.

**Fig. 4:**
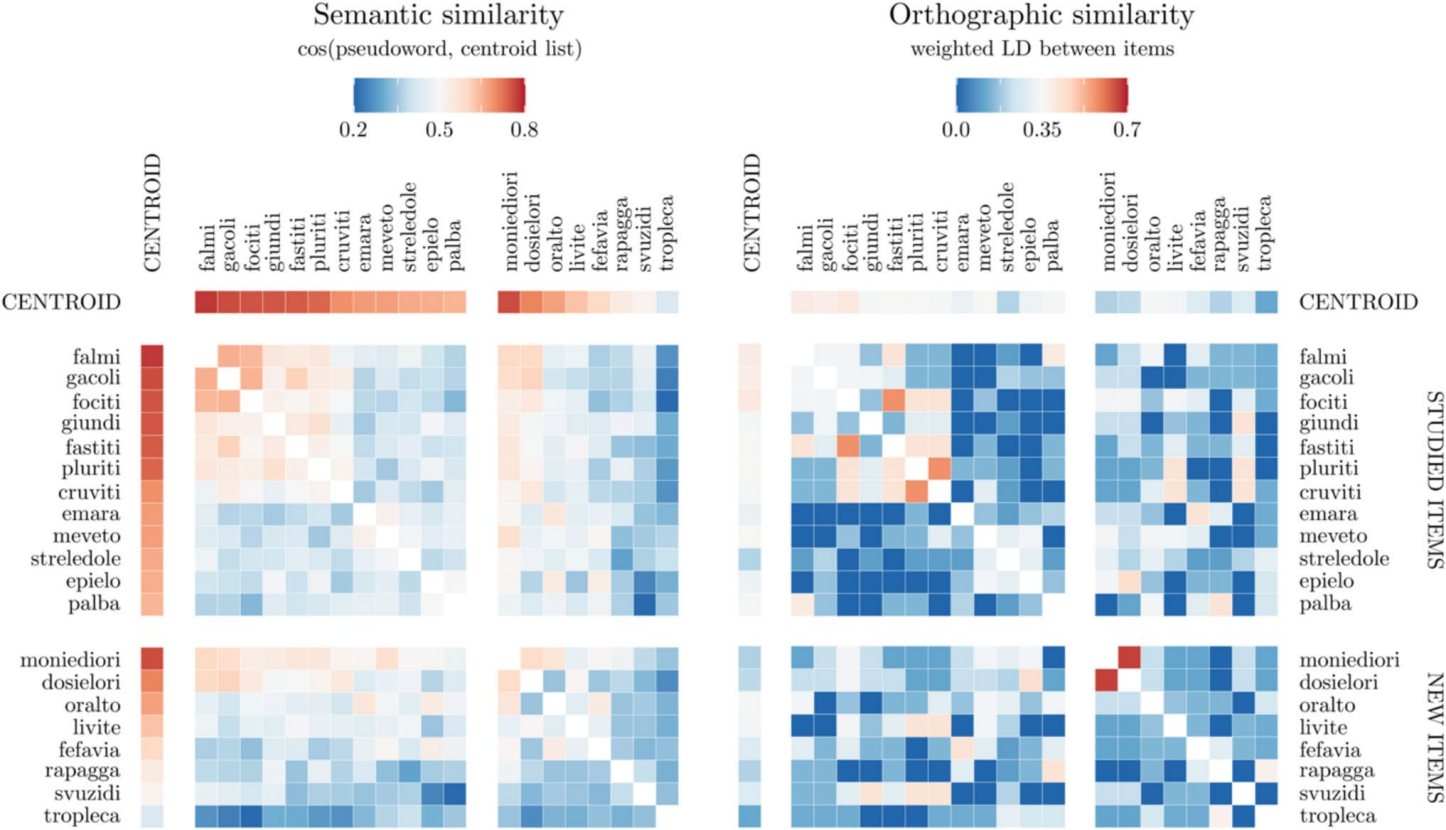
Heatmap representing the semantic (**left**) and orthographic (**right**) similarities among the items included in a sample list; warmer colors indicate higher similarities

The dependent variable was participants’ explicit responses in the recognition phase (“new” responses were scored as 0, “old” responses as 1). New and studied pseudowords were analyzed separately but following the same steps. Firstly, to control for possible confounding effects related to orthographical components we estimated as a baseline a multilevel logistic model including as a continuous predictor a measure of orthographic similarity. To compute this predictor, first, we computed the restricted Damerau-Levenshtein distance (LD^3^) between each item included in the recognition phase and those composing its list, second, we converted it into proximities ranging from 0 to 1 (i.e., by dividing the LD with the distance by the alignment length; see Osth & Zhang, 2024), and, third, we averaged such proximities at the item level. This index was finally subtracted from 1 to convert it into a similarity metrics. To test our hypotheses, we evaluated whether the model including semantic similarity as a continuous predictor was a better model as compared with the baseline one (see Fig. 4 for two heatmaps illustrating these two types of similarity). Notably, these two indexes were poorly correlated across both new, *r* = 0.28, and studied, *r* = 0.18, items.

**Fig. 5:**
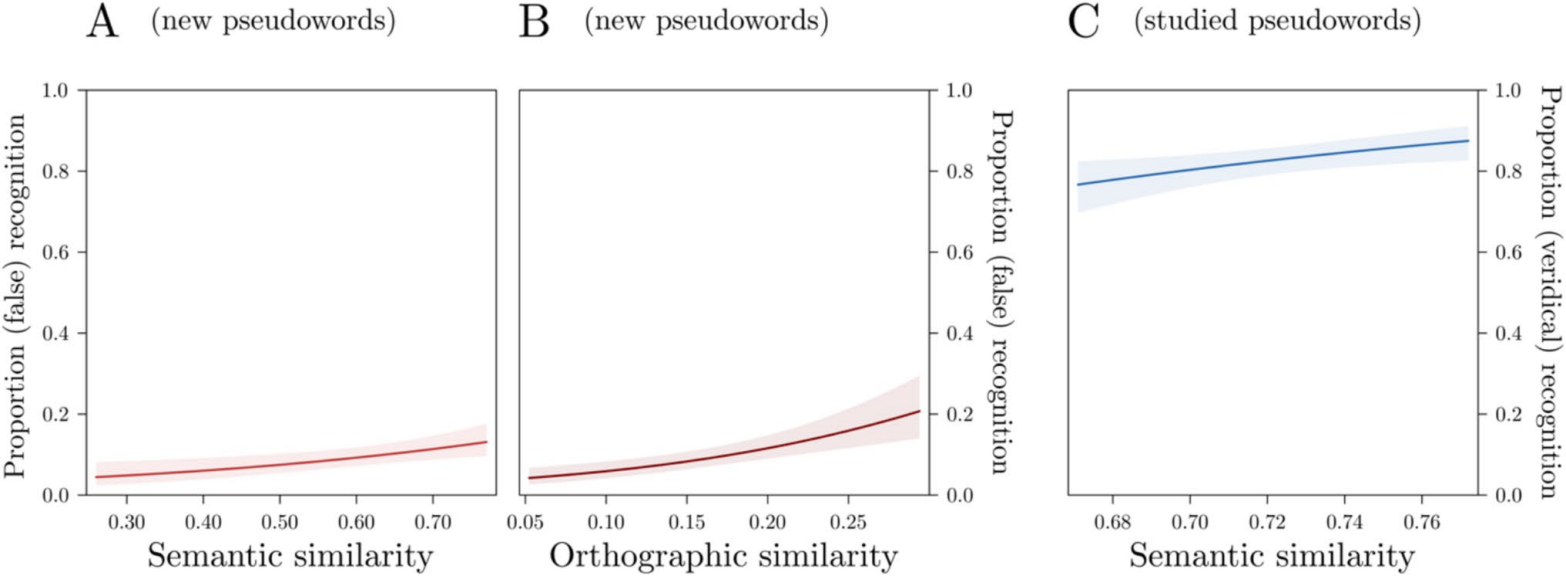
Plots of the results. Participants’ false (**A**, **B**) and veridical (**C**) recognitions increased at increasing semantic (**A**, **C**) and orthographic (**B**) similarity between the (new or studied) item and those included in the studied list

The orthographic model estimated on new pseudowords outperformed the random effects only model by 22.3 AIC points (AIC = 5492.9 and 5515.2, respectively) and showed that the effect of orthographic similarity was significant, *b* = 8.72, *z* = 5.19, *p* < 0.001. This indicates that false recognitions increased with increasing orthographic similarity between each new pseudoword and the items composing its list. Critically, the model including also semantic similarity (*marginal Pseudo-R*^*2*^ = 0.05; *total Pseudo-R*^*2*^ = 0.32) outperformed the orthographic one by 6 AIC points (AIC = 5486.9) and showed that the effect of semantic similarity was significant, *b* = 2.31, *z* = 2.88, *p* < 0.001 (Fig. 5A). This indicates that false recognitions increased at increasing estimated semantic similarity between each new pseudoword and the centroid of its list. The effect of orthographic similarity was significant in this latter model too, *b* = 7.36, *z* = 4.38, *p* < 0.001 (Fig. 5B).

The orthographic model estimated on studied pseudowords was outperformed by the random effects only model by 2 AIC points (AIC = 3568.6 and 3566.6, respectively). Conversely, the model including semantic similarity (*marginal Pseudo-R*^*2*^ = 0.01; *total Pseudo-R*^*2*^ = 0.18) outperformed the random effects only one by 2.4 AIC points (AIC = 3564.2) and showed that the effect of semantic similarity was significant, *b* = 7.46, *z* = 2.54, *p* = 0.01 (Fig. 5C). This indicates that veridical recognitions increased at increasing semantic similarity between each studied pseudoword and the centroid of its list.

## Discussion

In the present study we tested whether the semantic information evoked by pseudowords (conceived as bags of character n-grams) can trigger the DRM effect. Participants performed a classical DRM task but, instead of the typical familiar words, participants were asked to encode and recognize plausible (i.e., readable and orthotactically legal) but out-of-vocabulary strings of letters. Within this format, to build the stimuli composing each list we took advantage of a DSM, namely *fastText*, able to induce a semantic representation for unattested items based on the distributional properties of the n-grams embedded in them. Results showed that participants false and veridical recognition increased at increasing estimated semantic similarity between each stimulus and the stimuli comprising its list. Notably, the models including (also) semantic information outperformed the ones including only orthographic information, thus ruling out the possibility the effects observed were mainly driven by superficial perceptual components. This indicates in turn that humans rely on prior subword distributional information when processing novel verbal stimuli, offering insights into the (granular) structure of long-term semantic memory.

At a more basic level, these results extend well-established evidence indicating that semantic memory underlies false memory, with higher false recognition proportions for words semantically more related to those studied (e.g., Chang & Johns, 2023; Gatti et al., 2022; Petilli et al., 2024) and that individuals with higher semantic abilities do tend to make more false alarms (Gatti et al., 2024c). Similarly, it should be noted that, consistent with previous studies, here we observed stronger effects (on both the orthographic and the semantic sides) for new items as compared to studied ones (e.g., Gatti et al., 2022; Osth et al., 2020, 2024). However, for the task at hand, this conclusion should be drawn carefully since the distribution and the range of the predictor substantially differ across the two types of items, thus maximizing the possibility to observe an effect only for new items.

Interestingly, results from the baseline models showed that orthographic similarity predicts false recognitions for new pseudowords (in line with previous studies, Zeelenberg et al., 2005) but not veridical recognition for studied ones. To interpret this pattern, on the one hand we can infer that semantic information is not the only predictor of false alarms, but one of the major ones (also consistent with seminal results, e.g., Roediger et al., 2001). On the other hand, regarding the differential role of orthographic information for new vs. studied items, following our argumentation above, we have to clarify that the investigation of such effect was not our primary interest, and thus the stimuli construction (especially in terms of width of the distribution) was not optimal to test it. Future studies are required to answer this, possibly employing DRM lists in which orthographic information is ad hoc manipulated.

From a broader point of view, the present findings can be further framed within classical memory theories explaining human behavior in the DRM task. According to the fuzzy-trace theory (FTT; Brainerd & Reyna, 2002), participants would encode two different memory traces: a trace linked to episodic and perceptive features of the studied items, called verbatim trace, and a trace linked to the semantic content of each list, called gist trace, which would be responsible for the production of the false memories. Alternatively, according to the activation-monitoring framework (AMF; Roediger et al., 2001), the critical lure would be associatively hyperactivated by the presentation of the studied words (i.e., through spreading activation), with this leading to high levels of false recognitions. Within the FTT, the present findings are consistent with the semantic nature of the (gist) trace inducing false memories and thus indicate that subword information can participate to the construction of such a trace. On the other hand, since the AMF relies on the assumption that the associative network underlying spreading activation is composed of word nodes, these findings directly challenge (and possibly counter) such theoretical framework. That is, the cornerstone of the present work is the use of pseudowords, which have – by definition – no place in a traditional (associative) network (e.g. Collins & Loftus, 1975) and the finding that the subword semantic information elicited by pseudowords can trigger the DRM effect counters one of the basic assumptions of the AMF (i.e., that the DRM effect is rooted in associative processes at the word level, since the lists are created from free association norms; e.g., Stadler et al., 1999). Within this context, our findings are also consistent with previous studies employing DRM lists composed by pseudowords arranged based on more surface-level similarity (i.e., orthographic) and showing that the (orthographically) more similar critical pseudowords are falsely recognized more often (Zeelenberg et al., 2005). Taking this more broadly, the present findings indicate the need to take into account subword information within the associative network, thus refusing the concept of words as atomic units.

The fact that humans can falsely remember items that were never part of their language experience and the fact that similarity between new and studied items plays a role in the observed effects offer some insights about what a false memory is. As discussed above, pseudowords have no place in an associative network, and thus it sounds implausible that participants are activating new pseudowords via (word-level) associative processing when studying the DRM list. To find a solution to the conundrum, our findings can be framed within global matching models of recognition memory (e.g., SAM, Gillund & Shiffrin, 1984; MINERVA2, Hintzman, 1988; TODAM, Murdock, 1982; or REM, Shiffrin & Steyvers, 1997; for a review, see Osth & Dennis, 2024; but see also Chang et al., in press, for recent integrations between MINERVA2 and DRM dual-traces theories). Indeed, these models trace back false memory to similarity-based matching processes taking place during the recognition phase. That is, during recognition tasks, humans would match the test item(s) against stored memory traces based on global similarity rather than strict identity. This purely reconstructive process would then underlie false memory. The present results are in line with the predictions of these models and further support the conclusion that false memory formation is a phenomenon occurring during retrieval rather than encoding.

Finally, our results can be traced back to humans’ tendency to detect systematic and statistical regularities in the (language) environment (Romberg & Saffran, 2010; Vidal et al., 2021) and thus to non-arbitrary perspectives on language (Dingemanse et al., 2015). More importantly, these findings extend previous evidence indicating that humans are sensitive to the semantic patterns elicited by novel words (e.g., Gatti et al., 2023a, 2024a, 2024b) by showing that this sensitivity is so profound and remarkable that it can even induce participants to falsely recognize stimuli that they never encountered in their entire lives.

In conclusion, using DSMs we provide evidence that humans are able to exploit subword information when dealing with novel words in the DRM task, thus demonstrating that semantic patterns evoked by pseudowords can trigger this well-established false memory effect. Our findings directly support theories on the non-arbitrariness of language and provide novel insights into the distributed structure of human semantic and false memory.

## Data Availability

All data, scripts and codes used in the analysis are available from the Open Science Framework at: https://osf.io/rv3fe/?view_only=9d09d9fc6c634d0ea31a1457f0ebe235. This study was not preregistered.

## Footnotes

1 It should also be noted that, since *fastText* hashes n-grams into bins, including the ones that were not observed in the training data, untrained n-grams (i.e., those that do not belong to a word attested in the training corpus) will be mapped to a random vector (or to the vector corresponding to another n-gram in case of a collision).

2 Because *fastText* is based on very large natural language corpora and might have ended up including some non-existent string by mistake, we systematically checked whether a “whole-pseudoword” vector was available in the corpus for the pseudowords included. In such cases, indeed, *fastText* could learn distributional patterns about these pseudowords as if they were meaningful elements, even if their occurrence was based on errors and typos. No full vector representation was available for all the pseudowords included in this study.

3 In this case, LD is defined as the minimum number of single-character edits required to change one element into the other, counting transposition of adjacent characters as 1; see Zhang and Osth (2024) for the motivation for this method in recognition memory tasks.

## Declarations

### Prior dissemination

A previous version of this article was uploaded on PsyArXiv (https://osf.io/preprints/psyarxiv/8um2f)

## References

[CR1] Bartlett, F. C. (1932). *Remembering*. Cambridge University Press.

[CR2] Bates, D., Mächler, M., Bolker, B., & Walker, S. (2015). Fitting linear mixed-effects models using lme4. *Journal of Statistical Software,**67*(1), 1–48.

[CR3] Bojanowski, P., Grave, E., Joulin, A., & Mikolov, T. (2017). Enriching word vectors with subword information. *Transactions of the Association for Computational Linguistics,**5*, 135–146.

[CR4] Bonandrini, R., Amenta, S., Sulpizio, S., Tettamanti, M., Mazzucchelli, A., & Marelli, M. (2023). Form to meaning mapping and the impact of explicit morpheme combination in novel word processing. *Cognitive Psychology,**145*, 101594. 37598658 10.1016/j.cogpsych.2023.101594

[CR5] Brainerd, C. J., & Reyna, V. F. (2002). Fuzzy-trace theory and false memory. *Current Directions in Psychological Science,**11*(5), 164–169. 10.1177/0963721415588081 PMC4651171 26594099

[CR6] Brainerd, C. J., Chang, M., & Bialer, D. M. (2020). From association to gist. *Journal of Experimental Psychology: Learning, Memory, and Cognition,**46*(11), 2106. 32658546 10.1037/xlm0000938

[CR7] Brainerd, C. J., Yang, Y., Reyna, V. F., Howe, M. L., & Mills, B. A. (2008). Semantic processing in “associative” false memory. *Psychonomic Bulletin & Review,**15*, 1035–1053. 19001566 10.3758/PBR.15.6.1035

[CR8] Cassani, G., Chuang, Y. Y., & Baayen, R. H. (2020). On the semantics of nonwords and their lexical category. *Journal of Experimental Psychology: Learning, Memory, and Cognition,**46*(4), 621. 31318232 10.1037/xlm0000747

[CR9] Chang, M., & Johns, B. (2023). Integrating Distributed Semantic Models with an Instance Memory Model to Explain False Recognition. In *Proceedings of the Annual Meeting of the Cognitive Science Society* (Vol. 45, No. 45).

[CR10] Chang, M., Johns, B. T., & Brainerd, C. J. (in press). True and False Recognition in MINERVA2: Integrating Fuzzy-Trace Theory and Computational Memory Modeling. *Psychological Review*. 10.1037/rev0000541 40014530

[CR11] Chuang, Y. Y., Vollmer, M. L., Shafaei-Bajestan, E., Gahl, S., Hendrix, P., & Baayen, R. H. (2021). The processing of pseudoword form and meaning in production and comprehension: A computational modeling approach using linear discriminative learning. *Behavior Research Methods,**53*, 945–976. 32377973 10.3758/s13428-020-01356-w PMC8219637

[CR12] Coane, J. H., McBride, D. M., Huff, M. J., Chang, K., Marsh, E. M., & Smith, K. A. (2021). Manipulations of list type in the DRM paradigm: A review of how structural and conceptual similarity affect false memory. *Frontiers in Psychology,**12*, 668550. 34135826 10.3389/fpsyg.2021.668550 PMC8200635

[CR13] Collins, A. M., & Loftus, E. F. (1975). A spreading-activation theory of semantic processing. *Psychological Review,**82*(6), 407.

[CR14] Deese, J. (1959). On the prediction of occurrence of particular verbal intrusions in immediate recall. *Journal of Experimental Psychology,**58*(1), 17. 13664879 10.1037/h0046671

[CR15] Dingemanse, M., Blasi, D. E., Lupyan, G., Christiansen, M. H., & Monaghan, P. (2015). Arbitrariness, iconicity, and systematicity in language. *Trends in Cognitive Sciences,**19*(10), 603–615. 26412098 10.1016/j.tics.2015.07.013

[CR16] Gatti, D., Marelli, M., & Rinaldi, L. (2023a). Out-of-vocabulary but not meaningless: Evidence for semantic-priming effects in pseudoword processing. *Journal of Experimental Psychology: General,**152*(3), 851. 36174173 10.1037/xge0001304

[CR17] Gatti, D., Raveling, L., Petrenco, A., & Günther, F. (2024a). Valence without meaning: investigating form and semantic components in pseudowords valence. *Psychonomic Bulletin & Review*, 1–13. 10.3758/s13423-024-02487-3 PMC11543720 38565840

[CR18] Gatti, D., Rinaldi, L., Marelli, M., Mazzoni, G., & Vecchi, T. (2022). Decomposing the semantic processes underpinning veridical and false memories. *Journal of Experimental Psychology: General,**151*(2), 363. 34941346 10.1037/xge0001079

[CR19] Gatti, D., Rinaldi, L., Mazzoni, G., & Vecchi, T. (2024b). Semantic and episodic processes differently predict false memories in the DRM task. *Scientific Reports,**14*(1), 256. 38167871 10.1038/s41598-023-50687-z PMC10761856

[CR20] Gatti, D., Rodio, F., Rinaldi, L., & Marelli, M. (2024c). On humans’(explicit) intuitions about the meaning of novel words. *Cognition,**251*, 105882. 39024842 10.1016/j.cognition.2024.105882

[CR21] Gatti, D., Stagnitto, S. M., Basile, C., Mazzoni, G., Vecchi, T., Rinaldi, L., & Lecce, S. (2023b). Individual differences in theory of mind correlate with the occurrence of false memory: A study with the DRM task. *Quarterly Journal of Experimental Psychology,**76*(9), 2107–2121. 10.1177/17470218221135178 36245220

[CR22] Gillund, G., & Shiffrin, R. M. (1984). A retrieval model for both recognition and recall. *Psychological Review,**91*(1), 1. 6571421

[CR23] Grave, E., Bojanowski, P., Gupta, P., Joulin, A., & Mikolov, T. (2018). Learning word vectors for 157 languages. *arXiv preprint *arXiv:1802.06893.

[CR24] Günther, F., Rinaldi, L., & Marelli, M. (2019). Vector-space models of semantic representation from a cognitive perspective: A discussion of common misconceptions. *Perspectives on Psychological Science,**14*(6), 1006–1033. 31505121 10.1177/1745691619861372

[CR25] Harris, Z. (1954). *Distributional Structure. Word,**10*(2–3), 146–162.

[CR26] Hendrix, P., & Sun, C. C. (2021). A word or two about nonwords: Frequency, semantic neighborhood density, and orthography-to-semantics consistency effects for nonwords in the lexical decision task. *Journal of Experimental Psychology: Learning, Memory, and Cognition,**47*(1), 157. 31999159 10.1037/xlm0000819

[CR27] Hintzman, D. L. (1988). Judgments of frequency and recognition memory in a multiple-trace memory model. *Psychological Review,**95*(4), 528.

[CR28] Jones, M. N., Willits, J., Dennis, S., & Jones, M. (2015). Models of semantic memory. *Oxford Handbook of Mathematical and Computational Psychology,**1*, 232–254.

[CR29] Joosse, A. Y., Kuscu, G., & Cassani, G. (2024). You Sound Like an Evil Young Man: A Distributional Semantic Analysis of Systematic Form-meaning Associations for Polarity, Gender, and Age in Fictional Characters’ Names. *Journal of Experimental Psychology: Learning, Memory, & Cognition*. 10.1037/xlm0001345 39298239

[CR30] Joulin, A. (2016). Fasttext. zip: Compressing text classification models. *arXiv preprint *arXiv:1612.03651.

[CR31] Keuleers, E., & Brysbaert, M. (2010). Wuggy: A multilingual pseudoword generator. *Behavior Research Methods,**42*, 627–633. 20805584 10.3758/BRM.42.3.627

[CR32] Kumar, A. A. (2021). Semantic memory: A review of methods, models, and current challenges. *Psychonomic Bulletin & Review,**28*(1), 40–80. 32885404 10.3758/s13423-020-01792-x

[CR33] Mandera, P., Keuleers, E., & Brysbaert, M. (2017). Explaining human performance in psycholinguistic tasks with models of semantic similarity based on prediction and counting: A review and empirical validation. *Journal of Memory and Language,**92*, 57–78.

[CR34] Mikolov, T. (2013). Efficient estimation of word representations in vector space. *arXiv preprint *arXiv:1301.3781.

[CR35] Montefinese, M., Ambrosini, E., Fairfield, B., & Mammarella, N. (2014). The adaptation of the affective norms for English words (ANEW) for Italian. *Behavior Research Methods,**46*, 887–903. 24150921 10.3758/s13428-013-0405-3

[CR36] Murdock, B. B. (1982). A theory for the storage and retrieval of item and associative information. *Psychological Review,**89*(6), 609. 10.1037/0033-295x.100.2.183 8483981

[CR37] Osth, A. F., & Dennis, S. (2024). Global Matching Models of Recognition Memory. In The Oxford *Handbook of Human Memory, Two Volume Pack: Foundations and Applications* (pp. 895–922). Oxford University Press.

[CR38] Osth, A. F., & Zhang, L. (2024). Integrating word-form representations with global similarity computation in recognition memory. *Psychonomic Bulletin & Review,**31*(3), 1000–1031. 37973762 10.3758/s13423-023-02402-2 PMC11192833

[CR39] Osth, A. F., Shabahang, K. D., Mewhort, D. J., & Heathcote, A. (2020). Global semantic similarity effects in recognition memory: Insights from BEAGLE representations and the diffusion decision model. *Journal of Memory and Language,**111*, 104071.

[CR40] Peirce, J., Gray, J. R., Simpson, S., MacAskill, M., Höchenberger, R., Sogo, H., Kastman, E., & Lindeløv, J. K. (2019). PsychoPy2: Experiments in behavior made easy. *Behavior Research Methods,**51*(1), 195–203. 30734206 10.3758/s13428-018-01193-y PMC6420413

[CR41] Petilli, M. A., Marelli, M., Mazzoni, G., Marchetti, M., Rinaldi, L., & Gatti, D. (2024). From Vector Spaces to DRM lists: False Memory Generator, a software for automated generation of lists of stimuli inducing false memories. *Behavior Research Methods,**56*(4), 3779–3793. 38710986 10.3758/s13428-024-02425-0 PMC11133058

[CR42] Roediger, H. L., & McDermott, K. B. (1995). Creating false memories: Remembering words not presented in lists. *Journal of Experimental Psychology: Learning, Memory, and Cognition,**21*(4), 803.

[CR43] Roediger, H. L., Watson, J. M., McDermott, K. B., & Gallo, D. A. (2001). Factors that determine false recall: A multiple regression analysis. *Psychonomic Bulletin & Review,**8*, 385–407. 11700893 10.3758/bf03196177

[CR44] Romberg, A. R., & Saffran, J. R. (2010). Statistical learning and language acquisition. *Wiley Interdisciplinary Reviews: Cognitive Science,**1*(6), 906–914. 21666883 10.1002/wcs.78 PMC3112001

[CR45] RStudio Team. (2015). *RStudio: Integrated development for R*. RStudio Inc. http://www.rstudio.com/

[CR46] Schütze, H. (1992). Word space. *Advances in Neural Information Processing Systems,**5*, 895–902.

[CR47] Shiffrin, R. M., & Steyvers, M. (1997). A model for recognition memory: REM—retrieving effectively from memory. *Psychonomic Bulletin & Review,**4*, 145–166. 21331823 10.3758/BF03209391

[CR48] Stadler, M. A., Roediger, H. L., & McDermott, K. B. (1999). Norms for word lists that create false memories. *Memory & Cognition,**27*, 494–500. 10355238 10.3758/bf03211543

[CR49] Sulin, R. A., & Dooling, D. J. (1974). Intrusion of a thematic idea in retention of prose. *Journal of Experimental Psychology,**103*(2), 255.

[CR50] Venables, W. N., & Ripley, B. D. (2002). *Modern applied statistics with S* (4th ed.). Springer.

[CR51] Vidal, Y., Viviani, E., Zoccolan, D., & Crepaldi, D. (2021). A general-purpose mechanism of visual feature association in visual word identification and beyond. *Current Biology,**31*(6), 1261–1267. 33417881 10.1016/j.cub.2020.12.017

[CR52] Wittgenstein, L. (1953). *Philosphical investigations*. MacMillan.

[CR53] Zeelenberg, R., Boot, I., & Pecher, D. (2005). Activating the critical lure is unnecessary for false recognition. *Consciousness & Cognition,**14*, 316–326. 15950885 10.1016/j.concog.2004.08.004

[CR54] Zhang, L., & Osth, A. F. (2024). Modelling orthographic similarity effects in recognition memory reveals support for open bigram representations of letter coding. *Cognitive Psychology,**148*, 101619. 38043466 10.1016/j.cogpsych.2023.101619

